# Creating High-Resolution Microscopic Cross-Section Images of Hardwood Species Using Generative Adversarial Networks

**DOI:** 10.3389/fpls.2021.760139

**Published:** 2021-10-13

**Authors:** Dercilio Junior Verly Lopes, Gustavo Fardin Monti, Greg W. Burgreen, Jordão Cabral Moulin, Gabrielly dos Santos Bobadilha, Edward D. Entsminger, Ramon Ferreira Oliveira

**Affiliations:** ^1^Department of Sustainable Bioproducts, Forest and Wildlife Research Center, Mississippi State University, Starkville, MS, United States; ^2^Universidade Federal do Espírito Santo, Centro Universitario do Norte do Espírito Santo, São Mateus, Brazil; ^3^Center for Advanced Vehicular Systems, Mississippi State University, Starkville, MS, United States; ^4^Departamento de Ciências Florestais e da Madeira, Universidade Federal do Espírito Santo, Jerônimo Monteiro, Brazil

**Keywords:** wood anatomy, machine learning, artificial intelligence, wood image transformation, microscopic images, StyleGAN

## Abstract

Microscopic wood identification plays a critical role in many economically important areas in wood science. Historically, producing and curating relevant and representative microscopic cross-section images of wood species is limited to highly experienced and trained anatomists. This manuscript demonstrates the feasibility of generating synthetic microscopic cross-sections of hardwood species. We leveraged a publicly available dataset of 119 hardwood species to train a style-based generative adversarial network (GAN). The proposed GAN generated anatomically accurate cross-section images with remarkable fidelity to actual data. Quantitative metrics corroborated the capacity of the generative model in capturing complex wood structure by resulting in a Fréchet inception distance score of 17.38. Image diversity was calculated using the Structural Similarity Index Measure (SSIM). The SSIM results confirmed that the GAN approach can successfully synthesize diverse images. To confirm the usefulness and realism of the GAN generated images, eight professional wood anatomists in two experience levels participated in a visual Turing test and correctly identified fake and actual images at rates of 48.3 and 43.7%, respectively, with no statistical difference when compared to random guess. The generative model can synthesize realistic, diverse, and meaningful high-resolution microscope cross-section images that are virtually indistinguishable from real images. Furthermore, the framework presented may be suitable for improving current deep learning models, helping understand potential breeding between species, and may be used as an educational tool.

## Introduction

Transverse microscopic cross-sections of wood species have long been used for forensic wood identification, for analysis of critically important properties such as permeability and treatability with chemical agents, and to gain an understanding of the functioning of the tree (Zhang and Cai, 2006; Esteves and Pereira, 2008; Martins et al., 2013; Leggate et al., 2020; Lengowski et al., 2020; Słupianek et al., 2021). Microscopic capture of various anatomical features is accomplished in the lab by preparing individual thin slices of wood samples through standard stringent procedures that include several manually intensive steps: softening, cutting, clearing, staining, dehydrating, and mounting of the thin wood sections (Jansen et al., 1998).

Historically, creating and curating large datasets of microscopic wood images has been cumbersome with only a handful of datasets available to the public for research and development. The dataset produced by Martins et al. (2013) is perhaps the most used dataset for benchmarking several different wood identification approaches. The art of wood identification using such datasets is limited to only highly trained and experienced wood anatomists, due to the complexity of the wood structure within species and among a multitude of different species. Moreover, the number of senior wood anatomists with broad taxonomic expertise is declining (Lens et al., 2020). These limitations have set the stage for new artificial intelligence/machine-learning (AI/ML) technologies to make significant advances into the wood identification process.

Currently, deep learning in the form of convolutional neural networks (CNN) and optimization algorithms is beginning to revolutionize wood identification services. In fact, this technology is matching or surpassing expert wood anatomists in both macroscopic and microscopic image recognition and is being increasingly proposed as an adjunct to human wood identification decision-making (Hafemann et al., 2014; Lens et al., 2020; Lopes et al., 2020, 2021; Olschofsky and Köhl, 2020; de Geus et al., 2021; Fabijańska et al., 2021). The growth of computer-based wood identification and many other recognition tasks is facilitated by recent advancements in computational power, especially with graphical processing units (GPUs), which have enabled the widespread use of supervised machine-learning.

The AI/ML approaches have a rich potential within wood science and technology. For example, computer vision approaches could help identify and protect forests in the future (Lens et al., 2020). In this case, the expansion of computer vision-based wood identification would heavily depend on either establishing traditional extensive collaborations across wood science organizations as explained by Hwang and Sugiyama (2021) or through the development and application of artificial intelligence solutions that are novel, economically relevant, innovative, and stakeholder-engaged.

Successful applications of deep learning for wood identification are based on supervised learning algorithms that critically depend on labeled data for training purposes (Hwang and Sugiyama, 2021). For example, Martins et al. (2013); Filho et al. (2014), and Hafemann et al. (2014) applied deep CNN models on macroscopic and microscopic images by manually labeling the forest wood species. Their custom deep learning-based model achieved 96.0 and 97.0% accuracies on the macroscopic and microscopic datasets, respectively. Similarly, Fabijańska et al. (2021) automatically identified 14 European tree species using a residual convolutional encoder network in a sliding window with 99.0% accuracy. Collecting large sets of labeled training data constitutes a non-trivial bottleneck in AI/ML workflows. However, AI/ML has the potential to artificially synthesize the requisite labeled data, which we will explore in this manuscript.

Generative adversarial networks (GANs) are special types of deep learning where two neural networks are trained simultaneously, with the generator Network G, focusing on image generation from feedback given by a discriminator Network D, that is designed to determine whether a given input data is from an actual dataset or is synthetically generated (fake) by G (Yi et al., 2019). The GANs can achieve state-of-the-art synthetic generation of remarkably realistic images using CNN in an unsupervised manner. The GANs have been successfully applied in many fields including medical analysis, satellite imagery, computational fluid dynamics, and precision agriculture (Goodfellow et al., 2014; Nie et al., 2018; Wu et al., 2020; Pang et al., 2021).

Given the ability to use deep learning to synthesize images from multiple domains, we herein seek to explore the utility of GANs to map and generate labeled microscopic images on a large number of hardwood species. Therefore, the purpose of this manuscript is fourfold: (1) to demonstrate the feasibility of image synthesis in the field of wood anatomy; (2) to quantitatively and qualitatively assess the quality of generated images; (3) to present synthetically generated images to experts in the field through a visual Turing test (VTT); and (4) to raise awareness of the potential of deep learning techniques for steering the forestry and forest and wood products industry toward transformative directions.

To our knowledge, no study has been conducted using GANs to synthesize and critically evaluate microscopic cross-sectional images of hardwood species or in wood anatomy in general. This study seeks to demonstrate proof-of-concept technical and computational feasibility of performing image domain transformation to better equip wood anatomists and to introduce the wood science and technology communities to a novel AI/ML-based approach.

## Materials and Methods

### Transverse Microscopic Hardwoods Section Dataset

**FIGURE 1:**
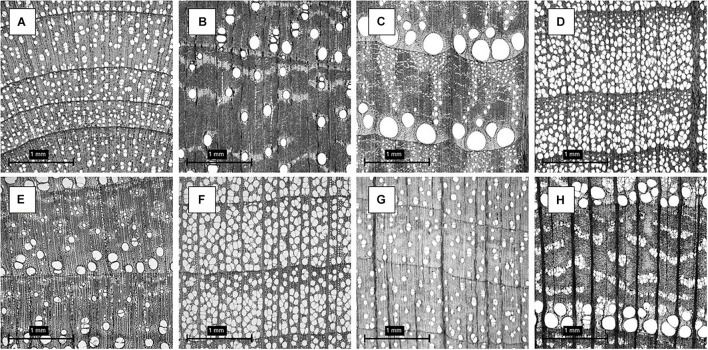
**(A)** Betulaceae - *Alnus firma*; **(B)** Cannabaceae - *Aphananthe aspera*; **(C)** Fagaceae - *Quercus crispula*; **(D)** Fagaceae - *Fagus japonica*; **(E)** Lauraceae - *Sassafras tzumu*; **(F)** Magnoliaceae - *Liriodendron tulipifera*; **(G)** Sapindaceae - *Acer distylum*; **(H)** Ulmaceae - *Ulmus laciniata*. Refer to the dataset for full dataset description.

This study was conducted using a publicly accessible transverse section of microscopic hardwood species dataset obtained from the Xylarium Digital Database (XDD) for Wood Information Science and Education – Kyoto University Research Information. This database was created, curated, processed, and labeled by Sugiyama et al. (2020). It was created in an effort to expand research and development in the area of wood anatomy and wood identification. The methods for obtaining the cross-section of the wood species are thoroughly described in the series of manuscripts published by the XDD research team in Hwang et al. (2018, 2020a, 2020b) and Kobayashi et al. (2019). Figure 1 shows eight different woody species present in the dataset.

Observing Figure 1, it is evident the diversity in anatomical structure with clear growth rings distinction, latewood and earlywood transitions, parenchyma cells, arrangement of parenchyma cells, fiber, vessel elements, pores and pores arrangement, multiple porosity classifications (ring, semi-ring, semi-diffuse, and diffuse porous), pore structure, and rays. These features are examples of key anatomical elements for hardwood identification. The full list of features and their terminology can be seen in Wheeler et al., 1989. The XDD dataset contained 7,051 images from 33 genus, 119 species, and 540 individuals at a resolution of 2.96 μm/pixel in a compressed HDF5 file at a grayscale resolution of 900 pixels × 900 pixels in JPEG (Joint Photographic Expert Group) format. The full description of the wood species can be seen in the Supplementary Material 1.

### Custom Training the Generative Adversarial Network

We leveraged the style-based generative adversarial network, henceforth StyleGAN model developed by Karras et al. (2019) to generate realistic microscopic cross-section images of hardwood species. The StyleGAN includes the progressive increase of resolution by adding layers to the network as described in Karras et al. (2018) with a series of later modifications described in Karras et al. (2019). The main reason for choosing StyleGAN was that it achieves state-of-the-art in human face transformations with extraordinary levels of detail. Similarly, to human faces, wood is a biological material with high-level attributes and stochastic variation in its structure, which requires an AI/ML framework that generates small and subtle intricacies of wood anatomy such as fibers, cells, pores shapes, pore arrangements, and rays, etc.

As the original image size was 900 pixels × 900 pixels, we resized the images to be 512 pixels × 512 pixels without further image processing. In this implementation, the StyleGAN progressively increased image size from 4^2^ pixels to 512^2^ pixels. We used 5,650 images for training. A latent vector of dimension 512 was used. The batch size decreased from 256 to 4 as training progressed. The adaptive momentum estimator (Adam) (Kingma and Ba, 2015) optimizer was used for training. The learning rate for the discriminator and generator were initially set to 0.0015 up to the resolution of 128^2^ pixels and slowly increased to 0.02 and 0.03 for resolutions of 256^2^ and 512^2^ pixels, respectively. The training setup doubled the image resolution when 600,000 images were shown to the discriminator. Training finished when the model had seen 7.5 million synthesized images. The Wasserstein GAN-gradient penalty (WGAN-GP) loss developed by Gulrajani et al. (2017) with modifications included by Karras et al. (2019) was used.. Throughout the training session, the model serialized checkpoints for later inference by using a script for image generation. The training took approximately 10 days. The computational resources used for this study included a workstation powered by 4 × NVIDIA GeForce RTX 2080Ti graphics processing units (GPU) with 11 GB of memory each and an Intel Core i9-9920K with a central processing unit (CPU) with 128 GB of memory.

### Quantitative Analysis of Generative Adversarial Network Images

There is no unified and universal metric to compare and evaluate generative adversarial networks (Borji, 2019). In the case of wood anatomy, the quantitative measure of GANs is limited or even non-existent. This work, to the best of our knowledge, is the first study to present GAN metrics in the domain of wood anatomy. For GAN metrics, we relied on the Fréchet inception distance (FID) by Heusel et al. (2018) and the Structural Similarity Index Measure (SSIM) by Hore and Ziou (2010) to assess the realism and diversity of the images generated by the StyleGAN.

The FID score is a metric that measures the maximum Gaussian entropy distribution for given mean and covariance. The difference of two Gaussians is then measured by Eq. 1:


(1) $$\text{FID}={||\mu_{r}-\mu_{g}||}^{2}+\text{TR}\,\left({\text{C}}_{r}+{\text{C}}_{g}-2\,{\left({\text{C}}_{r}\,{\text{C}}_{g}\right)}^{\frac{1}{2}}\right)$$

where, μ*_*r*_* and μ*_*g*_* and C*_*r*_* and C*_*g*_* are the mean and covariance of real and generated images.

The lower FID score means higher accuracy in synthetically generating microscopic cross-sectional images. The FID score enables a quantifiable anatomical comparison between a ground-truth image and a GAN generated image with respect to the fidelity of generated images.

The SSIM is a quality metric used to measure the similarity between two images. It is considered to be correlated with the quality perception of the human visual system (HVS) (Hore and Ziou, 2010). The SSIM is designed by modeling any image distortion as a combination of three factors, namely loss of correlation, luminance, and contrast distortions. The SSIM was defined by Eq. 2:


(2) SSIM⁢(f,g)=l⁢(f,g)⁢c⁢(f,g)⁢s⁢(f,g)

where,


(3) $$\text{l}\,\left(f,g\right)=\frac{2\,\mu_{f}\,\mu_{g}+{\text{C}}_{1}}{\mu_{f}^{2}+\mu_{g}^{2}+{\text{C}}_{1}}$$

(4) $$\text{c}\,\left(f,g\right)=\frac{2\,\sigma_{f}\,\sigma_{g}+{\text{C}}_{2}}{\sigma_{f}^{2}+\sigma_{g}^{2}+{\text{C}}_{2}}$$

(5) $$\text{s}\,\left(f,g\right)=\frac{\sigma_{\mathrm{fg}}+{\text{C}}_{3}}{\sigma_{f}\,\sigma_{g}+{\text{C}}_{3}}$$

Equations 3–5, respectively, refer to the luminance comparison function that measures the closeness of two images mean luminance (μ*_*f*_* and μ*_*g*_*); the contrast comparison function, which calculates the closeness of the contrast of the two images by the standard deviation (σ*_*f*_* and σ*_*g*_*); and the structure comparison function that measures the correlation coefficient between the two images, *f* and *g*. The σ*_*fg*_* argument is the covariance between *f* and *g*. A value of zero (0) means no correlation between images, and a value of one (1) means that *f* = *g* (Hore and Ziou, 2010).

### Visual Turing Test

To compare between actual and generated microscopic cross-section images of hardwood species, we used a VTT based on Park et al. (2021) and Chuquicusma et al. (2018). Our VTT experiments were conducted by a group of eight wood anatomy experts divided into two levels of expertise for analysis of microscopic wood images, namely, four intermediate wood anatomy experts [more than 1 and less than 5 years of experience (Group I)], and four advanced wood anatomy experts [more than 5 years of experience (Group II)].

The wood anatomists were blinded to each other’s evaluations of experiments and were not shown real or generated images prior to the experiments. The VTT contained 60 distinct 512^2^ images (30 actual images and 30 generated images). We randomly selected the images from the actual dataset, such that a minimum of three images were selected from each family. To avoid any bias, the generated image data were automatically generated by the StyleGAN. Furthermore, these images were not individually selected by our group.

The experts were given two choices to classify the fidelity of the images, namely, actual image or generated image. A website (Google Forms) was created to upload the images in a random manner. The link for the website can be seen in the GitHub.^1^ The visuals evaluated did not contain any information about the wood species and only the microscopic cross-section of hardwood species was presented.

In this experiment, the experts were not informed how many of the images were real or not real. The non-disclosed ratio allowed the evaluation of three important metrics: (1) number of incorrectly identified actual images (a high number represents how real the generated images look), (2) number of corrected identified real images (a high number represents how accurately the experts recognized salient anatomical features), and (3) a confusion metric that represents how effective our results were to confuse experts in identifying actual versus generated images.

The mean sensitivity, specificity, and accuracy of the eight expert VTT evaluations were calculated by Eqs 6–8.


(6) $$\mathrm{Sensitivity}=\frac{\mathrm{True}\,\text{positive}}{\left(\mathrm{True}\,\mathrm{positive}+\mathrm{False}\,\text{negative}\right)}$$

(7) $$\mathrm{Specificity}=\frac{\mathrm{True}\,\text{negative}}{\left(\mathrm{True}\,\mathrm{negative}+\mathrm{False}\,\text{positive}\right)}$$

(8) $$\mathrm{Accuracy}=\frac{\left(\mathrm{True}\,\mathrm{positive}+\mathrm{True}\,\text{negative}\right)}{\mathrm{Number}\,\mathrm{of}\,\text{observations}}$$

A statistical *t*-test was used to compare the means of the experts’ evaluations across the experiment. The scientific computing Python package Pauli et al. (2020) was used for the statistical analyses with the significance level set at *p* ≤ 0.05.

## Results and Discussion

### Feasibility of StyleGAN Generative Adversarial Network Training

**FIGURE 2:**
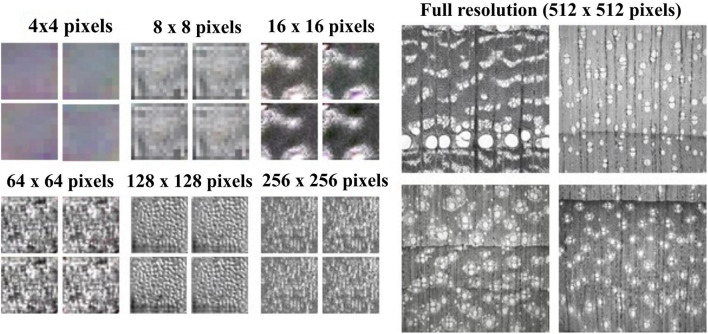
Overview of StyleGAN training using progressively increased image resolution from 4 × 4 pixels to 512 × 512 pixels.

The first goal of the study was to demonstrate the feasibility of training StyleGAN from scratch to generate realistic microscopic cross-section images of hardwood species. We found concomitant training improvement of the model up to approximately 7.5 million images seen by the discriminator, which corresponded to training at the final resolution of 512^2^ pixels. Figure 2 illustrates the progress of image generation as the resolution increased during training from 4^2^ to 512^2^ pixels. Initially, at 4^2^ pixels resolution, the generated images were pure abstract noise with concomitant progress in image quality with remarkable realism obtained at resolution of 512^2^ pixels. The StyleGAN trained as expected and was found to generate visually acceptable synthetic cross-section images of hardwood species.

### Qualitative Analysis of Generated Images

**FIGURE 3:**
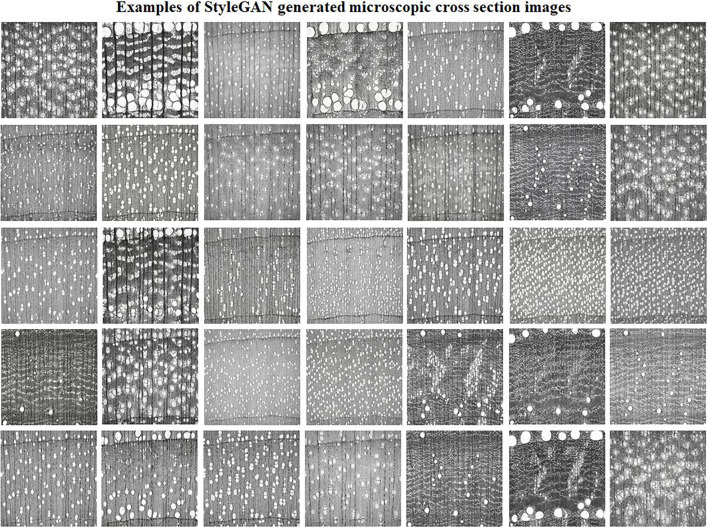
Examples of synthetic cross-section images of hardwood species produced by the StyleGAN.

Artificial intelligence and deep learning frameworks are revolutionizing interpretation, identification, and decision-making in wood species recognition. As data quantity and quality are critical to train deep learning-based image recognition systems, the proposed method herein should be useful to assist the computer vision wood identification community by providing realistic and meaningful microscopic images of cross-section of hardwood species. Using trained StyleGAN model, examples of the random generation of synthetic microscopic cross-section hardwood species are shown in Figure 3.

**FIGURE 4:**
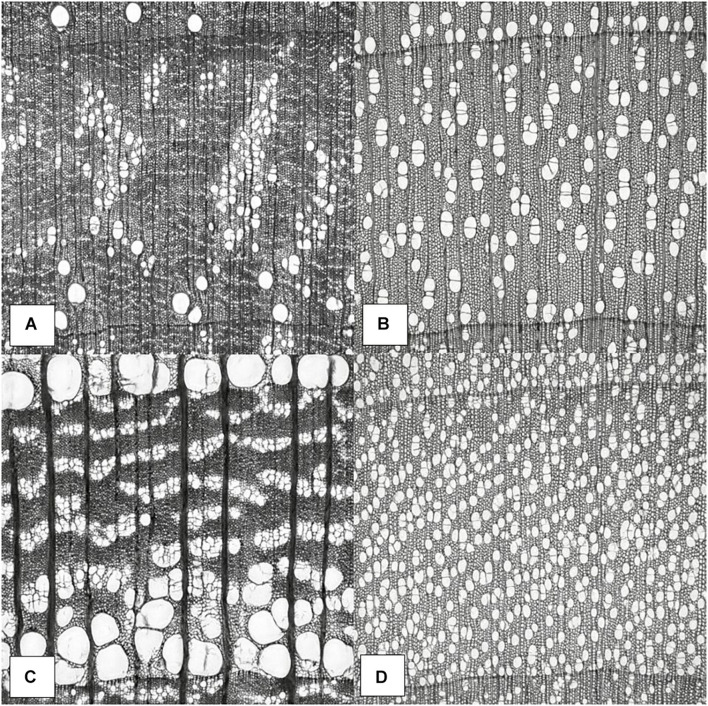
Wood anatomy images generated by StyleGAN. Synthesis of anatomical elements. Potential semi-ring-porous wood **(A)**, diffuse-porous wood **(B)**, ring-porous wood **(C)**, and diffuse-porous wood **(D)**.

Qualitatively, a remarkable variety of anatomical elements was generated by the trained generative adversarial StyleGAN network. The StyleGAN was capable of synthesizing high detail levels of the earlywood and latewood bands and growth ring transitions; ray width, height, and arrangement of apotracheal and paratracheal parenchyma cells; porosity such as ring-porous, semi-ring, semi-diffuse, and diffuse porous; and vessels with different arrangements and diameters were produced and recognized. Such detailed anatomical elements are what enable wood anatomists to scientifically identify wood species. Correct wood identification promotes reliable utilization of wood in various forms as in flooring, structural elements, plywood, particleboard, cross-laminated timber (CLT), various engineered wood products, and many other structural applications. Figure 4 illustrates the learned anatomical elements by the generative model in detail. Figures 4A–D should be carefully analyzed as these species do not exist, although may look similar to actual data. They were created using the StyleGAN generator, which allows control over various aspects of the image. They represent the capability of the proposed network in generating realistic and meaningful microscopic cross-section images of hardwood species.

In Figure 4A, there is a visible transition between earlywood and latewood growth ring bands, parenchyma and fiber cells noticeable, and uniseriate rays that are clearly seen. Different earlywood and latewood pores, pore arrangement, rays, initial earlywood band, and a few nested pores or pore clusters are also identifiable. There are also numerous solitary pores. No tyloses or mineral deposits can be seen in the vessel elements. The presence of paratracheal vasicentric, paratracheal aliform, and/or paratracheal confluent longitudinal parenchyma cells was not identifiable. Possible semi-ring-porous wood with clear separation between earlywood and latewood pores in Figure 4A.

In Figure 4B, the growth ring bands are visible, parenchyma and fiber cells noticeable, and uniseriate rays are clearly seen. There are numerous pore multiples that occur throughout, where two or more pores are connected to another pore. The radially arranged series of pore multiples or closely arranged solitary pores are visible as pore chains. These characteristics along with no clear separation between earlywood and latewood pores, small vessel element pore sizes, uniform pore size, and evenly distribution of the pores make this a possible diffuse-porous wood in Figure 4B.

In Figure 4C, shows a visible transition between earlywood and latewood growth ring bands, parenchyma and tracheids cells noticeable, and uniseriate rays are clearly seen in this cross-sectional view. Different earlywood and latewood pores, broad rays and pore arrangement, and initial earlywood band are observable. The pores are arranged in irregular concentric bands that are tangential in the earlywood are wavy bands (ulmiform pore arrangement). A few nested pores or pore clusters are also identifiable. Few tyloses can be seen in the vessel elements as well. A few solitary pores that do not touch any other pores are clearly seen. Possible ring-porous wood with clear separation between earlywood and latewood pores in Figure 4C.

In Figure 4D, the growth ring bands are visible, parenchyma cells noticeable, and uniseriate rays are evident. The growth ring boundary is clearly delineated by a line of marginal parenchyma as several cells thick of longitudinal parenchyma. There are numerous pore multiples that occur throughout, where two or more pores are connected to another pore. The radially arranged series of pore multiples or closely arranged solitary pores are visible as pore chains. These characteristics along with no clear separation between earlywood and latewood pores transitions, the small vessel element pore sizes, uniform pore size, and evenly distribution of the pores make this a possible diffuse-porous wood in Figure 4D.

The potential applicability of generative adversarial in wood science and technology is tremendous. As macroscopic cross-section datasets become publicly available for research and development, especially from tropical species, GANs can be trained to generate unlimited numbers of realistic cross-sections of endangered wood species listed by CITES (Convention on International Trade in Endangered Species of Wild Fauna and Flora). The synthetic and meaningful images could then be implemented to train, validate, and test current deep learning wood species recognition models. The methodology of this work could potentially eliminate economic and processing burdens in acquiring images of tropical species for machine-learning purposes. Furthermore, the GANs framework proposed herein is a logical step to increase collaboration among academia, research laboratories, local, state, and federal agencies, private sector, and the industry.

Another innovative use of the StyleGAN framework demonstrated in this work is to generate anatomical elements of a hybrid from two targeted parental species. The training of GAN on microscopic cross-section images from two parental species would potentially generate a hybrid species. The generated hybrid would then be validated by a real hybrid species. If the generated hybrid possesses relevant and accurate information, this technology could potentially steer a series of new research directions within the wood science and technology field, especially in breeding and genetics for estimating wood permeability, strength, density, and calculating the hydraulic potential of the tree trunk of a species that has not even been planted.

While the StyleGAN implementation appears to be very useful in creating realistic and meaningful microscopic cross-section images for more robust deep learning models and targeted biological engineering, it could also create content to facilitate training and education in wood anatomy. The realistic images could provide personalized interactions based upon an individual’s experience and areas of expertise. For students interested in anatomical elements, the GAN frameworks could provide new content that would help in training a new workforce faster and cheaper. In that case, this work has the capabilities of extending the wood anatomy and wood identification body beyond research and development.

### Quantitative Analysis of Generated Images

**FIGURE 5:**
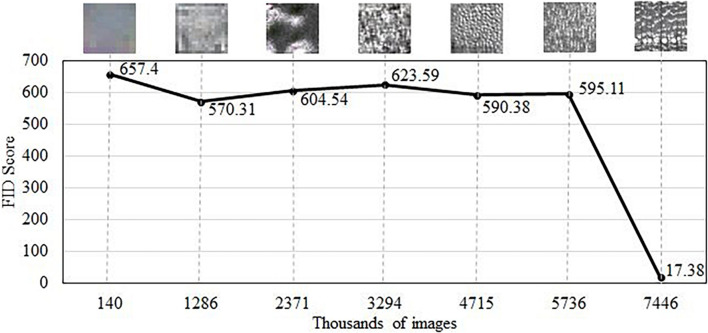
The Fréchet inception distance (FID) score achieved by the StyleGAN generative adversarial network (GAN) on cross-section images of hardwood species. Top images show the evolution of anatomical detail with training.

The FID score was calculated on 5,650 images drawn from the generator. The score was calculated by using the Inception-V3-network (Szegedy et al., 2016). The FID scores are reported in Figure 5. It was noted that as the model was trained, the StyleGAN model decreased the FID score from 657 points to a final value of 17.38, which indicates more realistic image quality generation at full resolution of 512^2^ pixels. The lower FID score of 17.38 means higher similarity between the two distributions, namely, between actual and synthetic data.

In the context of wood anatomy, it is not possible to compare the FID score to prior research or literature because this work is the first known application of generative adversarial for wood cross-sectional synthesis. However, GANs have been extensively used in different non-wood domains with comparatively low FID scores being reported. For instance, FID scores in Karras et al. (2019) were 4.40 for Flickr-Faces-HQ (FFHQ) on human faces, 2.65 for Large-scale Scene Understanding (LSUN) on a bedroom, and 3.27 for LSUN on car datasets, using an identical model. Conversely, in research by Skandarani et al. (2021), the FID scores were 24.74, 23.72, and 29.06 for cardiac, liver, and diabetic retinopathy datasets, respectively, also using StyleGAN. It is worth mentioning that the datasets used in Karras et al. (2019) were much larger than those in Skandarani et al. (2021) and in this work.

However, FID scores do not completely ensure reliability when evaluating diversity of image data (Borji, 2019). In order to further quantitatively assess the quality of our image synthesis, we calculated the structural similarity index for ground-truth pairs and ground-truth/generated image pairs on 5,650 actual and 5,650 generated images.

The XDD dataset used in this work consisted of hundreds of different species that would bring the SSIM to near zero (0.00) if the images were not correlated. The lower the SSIM, the more structurally different two given images are, which denotes diversity. To that end, the calculated SSIM for ground-truth training data pairs was 0.061 ± 0.015, which indicates a highly diverse dataset. Generally, collapsed GANs would generate similar images to the training set as explained by Srivastava et al. (2017); Lala et al. (2018), and Thanh-Tung and Tran (2020). In that case, the SSIM for ground-truth and generated images for collapsed GANs would be much higher, tending to approach 1.00. In this study, the calculated SSIM for the comparison between ground-truth and StyleGAN generated images was 0.061 ± 0.026. The intuition is relatively simple. The lower the SSIM, the more diverse the StyleGAN generated pairs seem to be. Likewise, Odena et al. (2017) used the same concept to evaluate the diversity of generated images from the ImageNet dataset. Furthermore, in this work, the StyleGAN model generated images as diverse as the training set, where the orange and blue curves highly overlapped (see Supplementary Material 2).

In order to provide a clear understanding about the StyleGAN implemented in this research, we developed a graphic user interface where one can generate images of microscopic hardwood species in a menu-driven and intuitive web application. The goal of this application is to provide knowledge about StyleGAN via user interactions. The application is an open-source framework available at https://github.com/LignumResearch/stylewood-model-usage. It is worth noting that the user has the capability of generating unlimited amount of data (images) with this pre-trained model.

### Anatomic Validation via Visual Turing Test

Table 1 summarizes the results of the realism assessment of images from the VTT by the eight wood anatomists. The mean accuracy obtained in the entire VTT was statistically lower than the random guessing [221/480 (46.04%) vs 240/480 (50.00%), respectively, *p* = 0.018]. In terms of correctly identifying generated images (specificity), there was no statistical difference between all wood anatomists and random guessing [116/240 (48.33%) vs 120/240 (50.00%), respectively, *p* = 0.6717]. Similarly, there was no statistical difference between all eight wood anatomists and random guessing to correctly identify actual images [105/240 (43.75%) vs 120/240 (50.00%), respectively, *p* = 0.064], despite the trend was in the predicted direction (*p* ≤ 0.05).

**TABLE 1:** Assessment of the realism of 60 images by the eight professional wood anatomists readers by the visual Turing test (VTT).

Group	Accuracy^a^ (%)	Sensitivity^b^ (%)	Specificity^c^ (%)

Group I^d^			
Wood Anatomist 02	50.0	43.3	56.7
Wood Anatomist 04	46.7	40.0	53.3
Wood Anatomist 05	46.7	40.0	53.0
Wood Anatomist 06	45.0	63.0	26.7

**Group II^e^**			

Wood Anatomist 01	41.7	43.3	40.0
Wood Anatomist 03	50.0	40.0	60.0
Wood Anatomist 07	40.0	36.7	43.3
Wood Anatomist 08	48.3	43.3	53.3

*^*a*^Overall mean [95% CI (confidence interval)] accuracy 46.1 (42.9–49.1).*

*^*b*^Overall mean (95% CI) sensitivity 43.7 (36.9–50.5).*

*^*c*^Overall mean (95% CI) specificity 48.3 (39.1–57.4).*

*^*d*^Group I: Wood anatomists with 1–5 years of experience. Mean (95% CI) accuracy 47.1 (43.8–50.4), sensitivity 46.7 (28.8–64.5), and specificity 47.5 (25.3–69.7).*

*^*e*^Group II: Wood anatomists with >5 years of experience. Mean (95% CI) accuracy 45.0 (37.2–52.8), sensitivity 40.8 (35.8–45.9), and specificity 49.15 (25.3–69.7).*

By analyzing Groups I and II, there was no statistical significant difference between the two groups for accuracy, sensitivity, and specificity, respectively [45.0 vs 47.1% (*p* = 0.548), 40.8 vs 46.6% (*p* = 0.317), and 49.2 vs 47.5% (*p* = 0.873)]. The only actual species captured (100% true positive) by all wood anatomists was *Litsea glutinosa*. Additionally, none of the wood anatomists (100% false negative) captured *Zelkova serrata*, which was also an actual species. The full data regarding the VTT can be obtained in the GitHub.

In summary, results of the VTT indicated that the StyleGAN synthetically generated image fidelity comparable to actual data. The VTT data suggests that the generated images were highly realistic and indistinguishable from real microscopic cross-section images of hardwood species, regardless of the level of expertise in anatomical evaluation.

## Conclusion

This study shows that StyleGAN can successfully synthesize highly realistic and anatomically meaningful 512^2^ microscopic cross-section images of hardwood species that are virtually indistinguishable from real cross-section images. We confirmed the realism and diversity for generated images by calculating the FID score, an SSIM distribution, and a VTT using two groups of professional wood anatomists with different levels of expertise.

We discussed several novel research directions involving wood anatomy and wood identification, StyleGAN, namely, data augmentation for current computer vision-based wood identification, dataset generation for wood species that are listed as threatened, endangered, or critical by CITES, and simulation of breeding between two parental woody species. Along with these applications, the StyleGAN can be used as an educational tool for improving training of a new workforce in wood anatomy and wood identification. It is our ultimate goal to provide AI/ML solutions that are reliable, economically relevant, safe, and robust to better equip the forestry and forest and wood products industries, students, researchers, staff, faculty, and enthusiasts in the field.

Future research will focus of exploring latent space when generating images. It would allow us to explore single attributes of a given species, for example porosity, ray thickness, growth ring, etc. to potentially increase model’s generalization. Specifically, this research would increase the meaning and realism of images and enable targeted effects on the generated images. Additionally, GANs can perform multimodal learning that enables image synthesis by feature description.

## Data Availability Statement

The datasets presented in this study can be found in online repositories. The names of the repository/repositories and accession number(s) can be found below: https://repository. kulib.kyoto-u.ac.jp/dspace/handle/2433/250016.

## Author Contributions

DL, JM, GSB, and EE established the scope of the project. DL and GB collected the dataset. DL and RO provided the context and contributed to the introduction. DL, JM, GSB, GB, GM, and RO prepared the dataset, implemented the machine learning pipeline, and analyzed the data. DL, GB, and EE wrote the manuscript. All authors read and approved the final manuscript.

## Conflict of Interest

The authors declare that the research was conducted in the absence of any commercial or financial relationships that could be construed as a potential conflict of interest.

## Publisher’s Note

All claims expressed in this article are solely those of the authors and do not necessarily represent those of their affiliated organizations, or those of the publisher, the editors and the reviewers. Any product that may be evaluated in this article, or claim that may be made by its manufacturer, is not guaranteed or endorsed by the publisher.

## Footnotes

1
https://github.com/LignumResearch/stylewood-model-usage
