# Supplementary figures and images for: Creating High-Resolution Microscopic Cross-Section Images of Hardwood Species Using Generative Adversarial Networks
Source: Front Plant Sci. 2021 Oct 13;12:760139. doi: 10.3389/fpls.2021.760139 (PMC8548738; doi:10.3389/fpls.2021.760139)

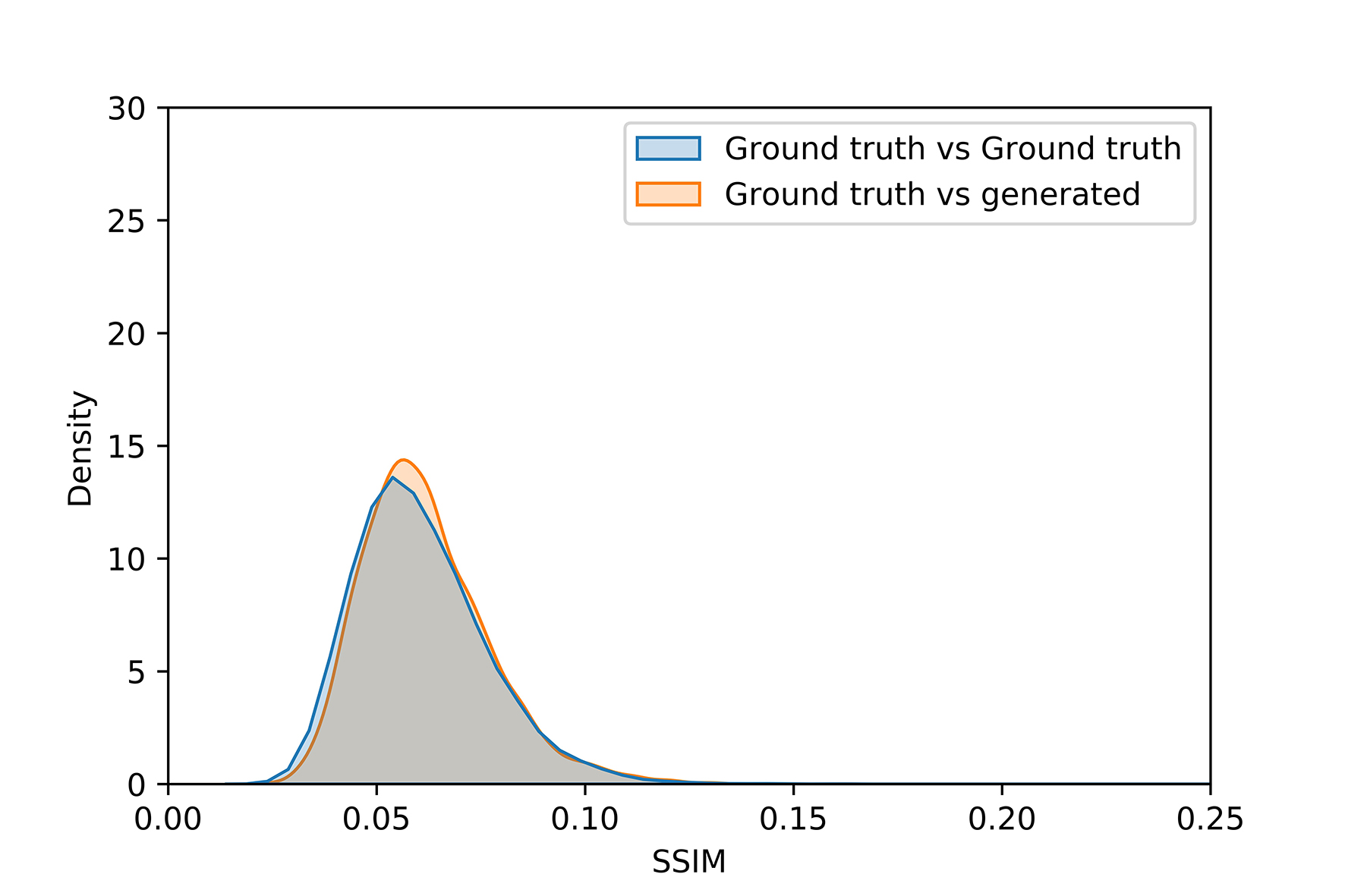

Supplement: Supplementary file 1 [file Image_1.JPEG]
