# Supplementary material for: Creating High-Resolution Microscopic Cross-Section Images of Hardwood Species Using Generative Adversarial Networks
Source: Front Plant Sci. 2021 Oct 13;12:760139. doi: 10.3389/fpls.2021.760139 (PMC8548738; doi:10.3389/fpls.2021.760139)
Supplement: Supplementary file 2 [file Table_1.DOCX]

Supplementary Material

# Supplementary Table

**Table 1.** Overview of the 119 woody species included in the Xylarium Digital Database (XDD) for Wood Information Science and Education – Kyoto University Research Information dataset.

| Family | Genus | Species |
| --- | --- | --- |
| Betulaceae | *Alnus* | *firma*  *hirsuta* var. *hirsuta*  *hirsuta* var. *sibirica*  *japonica* var. *japonica*  *matsumurae*  *pendula*  *sieboldiana*  *viridis* subsp. *maximowiczii* |
|  | *Betula* | *corylifolia*  *ermani*  *grossa*  *maximowicziana*  *platyphylla* var. *japonica* |
|  | *Carpinus* | *cordata*  *japonica*  *laxiflora*  *tschonoskii* |
|  | *Corylus* | *sieboldiana* |
|  | *Ostrya* | *japonica* |
| Cannabaceae | *Aphananthe* | *aspera* |
|  | *Celtis* | *sinensis* |
|  | *Trema* | *orientalis* |
| Fagaceae | *Castanea* | *crenata* |
|  | *Castanopsis* | *cuspidata*  *sieboldii* |
|  | *Fagus* | *crenata*  *japonica* |
|  | *Lithocarpus* | *edulis*  *glaber* |
|  | *Quercus* | *acuta*  *acutissima*  *crispula*  *dentata*  *gilva*  *myrsinifolia*  *phillyraeoides*  *salicina*  *serrata*  *variabilis* |
| Lauraceae | *Actinodaphne* | *acutivena*  *forrestii*  *mushaensis* |
|  | *Beilschmiedia* | *linocieroides* |
|  | *Cinnamomum* | *camphora*  *glanduliferum*  *longipetiolata*  *micranthum*  *porrectum*  *tenuipile*  *yabunikkei* |
|  | *Cryptocarya* | *chinensis* |
|  | *Laurus* | *nobilis* |
|  | *Lindera* | *communis*  *glauca*  *thomsonii*  *umbellata* |
|  | *Litsea* | *coreana*  *cubeba*  *elongata*  *glutinosa* |
|  | *Machilus* | *ichangensis*  *japonica*  *kusanoi*  *pauhoi*  *pingii*  *thunbergii*  *viridis*  *zuihoensis* |
|  | *Neolitsea* | *acuminatissima*  *acutotrinervia*  *cambodiana* |
|  | *Phoebe* | *lanceolata*  *macrocarpa*  *nanmu*  *neurantha*  *puwenensis*  *sheareri* |
|  | *Sassafras* | *tzumu* |
| Magnoliaceae | *Liriodendron* | *chinense*  *tulipifera* |
|  | *Magnolia* | *champaca*  *cylindrica*  *denudata*  *figo*  *fordiana* var. *forrestii*  *fordiana* var. *hainanensis*  *gioi*  *liliifora*  *lotungensis*  *macclurei*  *maudiae*  *maudiae* var. *platypetala*  *officinalis* var. *biloba*  *sargentiana*  *sprengeri*  *sumatrana* var. *glauca* |
| Sapindaceae | *Acer* | *amoenum*  *argutum*  *carpinifolium*  *crataegifolium*  *distylum*  *japonicum*  *micranthum*  *nipponicum*  *pictum* subsp*. dissectum*  *pictum* subsp. *dissectum f. connivens*  *pictum* subsp. *pictum f. ambiguum*  *rufinerve*  *sieboldianum* |
|  | *Aesculus* | *turbinata* |
|  | *Dodonaea* | *viscosa* |
|  | *Koelreuteria* | *paniculata* |
|  | *Sapindus* | *mukorossi* |
| Ulmaceae | *Ulmus* | *davidiana* var. *japonica*  *laciniata*  *parvifolia* |
|  | *Zelkova* | *serrata* |
